# Trends and implications for achieving VISION 2020 human resources for eye health targets in 16 countries of sub-Saharan Africa by the year 2020

**DOI:** 10.1186/1478-4491-12-45

**Published:** 2014-08-15

**Authors:** Jennifer J Palmer, Farai Chinanayi, Alice Gilbert, Devan Pillay, Samantha Fox, Jyoti Jaggernath, Kovin Naidoo, Ronnie Graham, Daksha Patel, Karl Blanchet

**Affiliations:** 1 International Centre for Eye Health, Clinical Research Department, Faculty of Infectious & Tropical Diseases, London School of Hygiene & Tropical Medicine, Keppel St, London WC1B 7HT, UK; 2 African Vision Research Institute, 172 Umbilo Road, Umbilo, Durban 4001, South Africa; 3 International Agency for the Prevention of Blindness (Africa Region), 172 Umbilo Road, Umbilo, Durban 4001, South Africa

**Keywords:** Human resources, Eye health, Sub-Saharan Africa, Cataract, Ophthalmology, Optometry, Nursing Vision 2020, Task-shifting

## Abstract

**Background:**

Development of human resources for eye health (HReH) is a major global eye health strategy to reduce the prevalence of avoidable visual impairment by the year 2020. Building on our previous analysis of current progress towards key HReH indicators and cataract surgery rates (CSRs), we predicted future indicator achievement among 16 countries of sub-Saharan Africa by 2020.

**Methods:**

Surgical and HReH data were collected from national eye care programme coordinators on six practitioner cadres: ophthalmologists, cataract surgeons, ophthalmic clinical officers, ophthalmic nurses, optometrists and ‘mid-level refractionists’ and combined them with publicly available population data to calculate practitioner-to-population ratios and CSRs. Data on workforce entry and exit (2008 to 2010) was used to project practitioner population and CSR growth between 2011 and 2020 in relation to projected growth in the general population. Associations between indicator progress and the presence of a non-physician cataract surgeon cadre were also explored using Wilcoxon rank sum tests and Spearman rank correlations.

**Results:**

In our 16-country sample, practitioner per million population ratios are predicted to increase slightly for surgeons (ophthalmologists/cataract surgeons, from 3.1 in 2011 to 3.4 in 2020) and ophthalmic nurses/clinical officers (5.8 to 6.8) but remain low for refractionists (including optometrists, at 3.6 in 2011 and 2020). Among countries that have not already achieved target indicators, however, practitioner growth will be insufficient for any additional countries to reach the surgeon and refractionist targets by year 2020. Without further strategy change and investment, even after 2020, surgeon growth is only expected to sufficiently outpace general population growth to reach the target in one country. For nurses, two additional countries will achieve the target while one will fall below it. In 2011, high surgeon practitioner ratios were associated with high CSR, regardless of the type of surgeon employed. The cataract surgeon workforce is growing proportionately faster than the ophthalmologist.

**Conclusions:**

The HReH workforce is not growing fast enough to achieve global eye health targets in most of the sub-Saharan countries we surveyed by 2020. Countries seeking to make rapid progress to improve CSR could prioritise investment in training new cataract surgeons over ophthalmologists and improving surgical output efficiency.

## Abstract (fr)

**Additional non-English language abstract (French):**

**Contexte**

Le développement des ressources humaines pour la santé oculaire est une priorité majeure des initiatives globales pour la réduction de la cécité d’ici à 2020. Sur la base de notre analyse précédente sur la situation en 2011, nous avons calculé les indicateurs de performance pour 2020 pour 16 pays de l’Afrique sub-saharienne.

**Méthodes**

Les données chirurgicales et en ressources humaines ont été collectées auprès des coordinateurs de programmes nationaux pour 6 cadres de santé (ophtalmologistes, opérateurs de la cataracte, techniciens supérieurs en ophtalmologie (TSO), infirmiers en ophtalmologie, optométristes et réfractionnistes) combinées avec les données démographiques. Les données sur entrée et sortie (2008–2011) de la force de travail ont été utilisées pour projeter le taux professionnel-population et le taux d’opérations chirurgicales entre 2011 et 2020. Les associations entre indicateur de performance et présence d’opérateurs de la cataracte ont été analysées en utilisant les tests de rang de somme Wilcoxon et les corrélations Spearman.

**Résultats**

Les taux professionnel-population vont augmenter légèrement pour les chirurgiens (ophtalmologistes/opérateurs de la cataracte, de 3,1 en 2011 à 3,4 en 2020) et les TSO/infirmier en ophtalmologie (5,8 à 6,8) mais restent bas pour les réfractionnistes (optométristes inclus, à 3,6). Parmi les pays qui ont déjà atteint les indicateurs cibles en 2011, la croissance actuelle sera insuffisante pour atteindre les cibles en termes de chirurgiens et réfractionnistes d’ici à 2020. Seul un pays peut atteindre la cible chirurgien après 2020. Pour les infirmiers, deux pays vont atteindre la cible en 2020 tandis qu’un troisième sera juste en dessous. En 2011, les taux importants en chirurgiens étaient associés avec de hauts taux d’opérations de chirurgie quel que soit le type de chirurgien employé. La population en opérateur de chirurgie augmente proportionnellement plus rapidement que celle d’ophtalmologistes.

La force de travail en santé oculaire n’augmente pas assez vite pour atteindre les cibles de performance d’ici à 2020 dans la plupart des pays d’Afrique sub-saharienne. Les pays qui veulent augmenter rapidement leur taux de chirurgie devraient investir sur les opérateurs de la cataracte plutôt que sur les ophtalmologistes, puisque cette population de professionnels peut s’accroitre plus rapidement.

## Background

Development of human resources for eye health (HReH) is one of the core pillars of the “VISION 2020: the Right to Sight” Global Initiative for the Elimination of Avoidable Blindness [1], and is recognised by the World Health Organization (WHO) as essential to achieving universal eye health by the year 2020 [2]. To achieve global eye health programme goals, the WHO Global Action Plan recommends monitoring key HReH indicators (practitioner per population ratios) thought to reflect the skills in a workforce necessary to address the main causes of avoidable visual impairment [2]. VISION 2020 strategies additionally recommend targets for these ratios which are specific to HReH cadre types and world regions [1]. One population-based service delivery output indicator is additionally suggested as a proxy indicator to measure the performance of eye health services in reducing avoidable visual impairment: annual cataract surgery rates (CSR, the number of cataract operations conducted per million population per year). Although the appropriateness and evidence supporting the selection of VISION 2020 targets is debated (Palmer and colleagues, manuscript submitted), many national and non-governmental eye health programmes use these as developmental benchmarks in practice.

In a previous paper (Palmer and colleagues, manuscript submitted), we presented estimates of current progress towards VISION 2020 HReH and cataract surgery targets in 21 countries of sub-Saharan Africa in 2011. Here, we expand on this analysis to predict whether countries in this sample will reach these suggested targets by the year 2020, given current workforce dynamics (particularly, workforce growth rates) and cadre mix (particularly, the presence of a non-physician cadre to share delivery of cataract surgery services).

Traditionally, programmes have focussed on training ophthalmologists to deliver cataract surgery. In some settings, due to a lack of ophthalmologists and general physicians, part of this task has been shifted to non-physician medical assistants/clinical officers and nurses. Since this cadre can be trained in a shorter time period and is typically easier to deploy and retain in rural areas, this human resource strategy is seen by some as an effective way to achieve VISION 2020 goals in Africa, although this is debated [3-7]. Correction of refractive errors is another key eye health service which can be shared in a convenient, cost-effective manner by both optometrists and a wide range of personnel such as ophthalmic assistants, opticians and other cadres [1].

Only one previous study has assessed global changes in HReH growth in relation to changes in patient populations to predict future need [8]. Using data on ophthalmologists entering and leaving the workforce in the year before the survey, these authors suggested that the global population of ophthalmologists was growing faster than the general population but slower than the population over 60 years of age who most need ophthalmic services. Scarcity of this type of information in Africa meant that they could draw no conclusions about how well the need for ophthalmic services will be met in the sub-Saharan region specifically. Information on growth of other HReH cadres has not so far been collected.

## Methods

### National questionnaires

National-level eye health services data were collected via electronic questionnaires circulated to key informants in all 33 countries of sub-Saharan Africa with more than 4 million population as of the year 2010, as well as in three countries less than 4 million population where research collaborations already existed (Botswana, Gambia, Guinea-Bissau). Sufficient data to include in analyses were received from 21 countries, giving an overall response rate of 58% (87% in Anglophone countries approached compared to 44% in Francophone, 33% in Horn of Africa and 0% in Lusophone) and representing a combined 2011 population of 633 million, or 72% of the population of sub-Saharan Africa. See Palmer and colleagues (manuscript submitted) for further details.

The questionnaire was designed to collect information on six HReH cadres, in three combined-cadre categories, identified in VISION 2020 plans and appropriate to the sub-Sahara African context:

1. ‘Surgeons’: considered to be (i) ophthalmologists (physicians (Medical Doctor or equivalent degree) who specialise in the eye and visual system) and (ii) cataract surgeons (generally non-physician ophthalmic nurses or clinical officers additionally trained in cataract surgery or, in two countries (Democractic Republic of Congo and Madagascar), non-ophthalmologist general physicians who conduct cataract surgery with limited training in ophthalmology).

2. ‘OCO/nurses’: (iii) ophthalmic clinical officers (OCOs)/medical assistants and (iv) ophthalmic nurses (both non-physician practitioners with an advanced (minimum 1 year) qualification in ophthalmology, including ‘techniciens supérieurs en ophtalmologie’ (TSOs) in Francophone countries).

3. ‘Refractionists’: (v) optometrists (personnel with Bachelor of Science or diploma in optometry (normally 3–4 years)) and (vi) mid-level refractionists (all other mid-level personnel with refraction training who perform refractions as a primary duty including refractionists, ophthalmic assistants/technicians, low vision specialists, opticians and equivalent).

HReH data was not collected on: community-based eye care educators, cataract case-finders, integrated eye care workers and equivalents, nor on non-ophthalmic surgical and inpatient ward staff, including general nurses or clinical officers with ‘on-the-job’ training in ophthalmology or refractive error services but without formal qualifications.

VISION 2020 HReH and cataract surgery targets are summarised in Table 1. For discussion on the history of VISION 2020 target selection, please see Palmer and colleagues (manuscript submitted).

**Table 1:** VISION 2020 human resources for eye health and cataract surgery targets for sub-Saharan Africa

	**VISION 2020 target per million population**
Ophthalmologists	4
Cataract surgeons	10
Ophthalmic clinical officers	
Ophthalmic nurses	
Optometrists	20
Mid-level refractionists	
Cataract surgery rate	2,000
Cataract surgeries per surgeon (surgical efficiency ratio)	500

In VISION 2020 documents, cataract surgeons, OCOs and ophthalmic nurses are combined as ‘mid-level personnel’ and share a single target [1]. In our analyses, we collected and reported separate data on all three cadres and combined cataract surgeons with ophthalmologists in a new ‘all surgeons’ category to highlight human resource needs associated with cataract surgical performance. Similarly, optometrists and mid-level refractionists were reported separately and combined in some analyses in an ‘all refractionists’ category.

The questionnaire was structured to collect HReH information in three parts, following the ‘working lifespan’ conceptual framework on workforce availability [9] and further informed by the WHO-AIMS questionnaire on mental health systems [10]:

1. Active workforce: practitioners currently in the workforce, including location (capital city or outside capital)

2. Entry into the eye care workforce: practitioners who were recently trained and recruited into the workforce

3. Exit from the workforce: practitioners who recently left the workforce

For all cadres in the active workforce, information was collected on the numbers of personnel in each cadre who entered (including recent graduates, immigrants, expatriates and those who re-entered the sector) and exited (due to retirement, death, emigration or other reason for leaving the country and temporary exit to pursue a new field of work) employment in the last 3 years to provide information for workforce projections. Whereas national programmes typically have reliable data on recent graduates, who represent the majority of people entering the workforce, information on workforce exit is typically poor. Coordinators were therefore encouraged to collate estimates on this variable that were provided by state- and district-level key informants using their personal, local knowledge.

For all categories of information collected, key informants were asked to provide data covering all sectors in the country (that is, government, non-governmental and private-for-profit).

When information on the number of cataract surgeries performed in 2011 could not be provided, informants were asked to provide data for another year (for example, 2010; non-2011 years are specified in Table and Figure footnotes). When surgery data could not be provided by informants in-country (Malawi and Zambia), the most recent estimate available in the International Agency for the Prevention of Blindness (IAPB) Africa database was used (Daniel Etya’ale, personal communication). In one country (Nigeria) reliable surgery data could only be provided from 13 out of 37 states; we therefore calculated a national estimate based on the proportional CSR in these states for use in all analyses.

While it was assumed that all surgeons (of both types) had been trained to perform cataract surgery and are thus theoretically capable of providing this service, no information was collected on the proportion of surgeons who are clinically active in the eye health workforce but who do not currently perform cataract surgery because of infrastructural limitations, strategic or other reasons. To provide some rough data on cadre-specific surgical efficiency (see below), national coordinators were asked to use personal local knowledge to estimate the proportion of cataract surgeries performed by ophthalmologists, since, in most countries, these data are not systematically collected.

### Data management and analysis

For consistency, all questionnaire data were reviewed by a single researcher to identify likely reporting errors requiring follow-up. Data were then entered directly into a Stata 12.1 (StataCorp, College Station, TX, USA; 2009) database and outputs cross-checked with questionnaires by a second researcher. Stata 12.1 was used for all statistical analyses described below. See Palmer and colleagues (manuscript submitted) for further details on analysis of current progress towards VISION 2020 targets. Analyses of the HReH workforce of individual countries are also available as additional files in that paper.

#### *National comparisons to VISION 2020 targets*

HReH practitioner per million population ratios were calculated to represent national and ‘regional’ (short-hand for all countries included in the sample, acknowledging that these countries may not be statistically representative of the entire sub-Saharan Africa region) HReH indicator performance in 2011. For all countries that reported complete data on practitioner entry and exit, the net change in practitioners in each cadre over the last 3 years (that is, total entered – total exited between 2008 and 2010) was then multiplied by three to project the number of practitioners expected in the workforce nine years from ‘now’ in the year 2020, assuming the current rate of growth (entry to and exit from the labour market) is maintained. These were compared to national population projections for the year 2020 ([11]; population estimates for South Sudan provided separately from the South Sudan National Bureau of Statistics, personal communication; Sudan projection estimated using 1.03% population growth rate) to predict practitioner per million population ratios in 2020.

Annual cataract surgeries performed per million population (that is, CSR) and per surgeon (that is, surgical efficiency ratio; considering ophthalmologists and cataract surgeons) were similarly calculated, using 2011 surgical data. To calculate CSRs in 2020, the projected number of cataract surgeries in 2020 was estimated by using cadre-specific rates of practitioner population increase multiplied by 2011 cadre-specific efficiency ratios of surgeries per surgeon. Surgical efficiency was assumed to be maintained at the 2011 rate throughout the 2011 to 2020 period (see Additional file three in Palmer and colleagues, manuscript submitted, for example calculations). HReH and surgical indicators at both time points were compared to VISION 2020 targets (Table 1). Countries were classified as improving if their practitioner workforce was proportionally increasing at a faster rate than the general country population (that is, (2020 population – 2011 population)/2011 population) such that their practitioner per million population ratio was expected to increase between 2011 and 2020. Proportional changes in HReH ratios over this period were plotted in relation to proportional VISION 2020 target achievement to compare predicted country progress graphically. Annual projected ratios between 2011 and 2050 were similarly estimated to identify how long after 2020 some countries are expected to reach VISION 2020 targets, at current rates of practitioner and projected general population growth. The same process to classify CSR trends was followed as for HReH ratios.

#### *Multi-country comparisons*

Current and projected HReH and CSR target performance across the sample was assessed in three ways: by pooling the total number of practitioners or surgeries from all countries and dividing this numerator by the total population of all countries reporting data (sub-Saharan Africa ‘regional ratio’); by calculating the average practitioner-to-population ratio and CSR across countries (country mean); and by calculating the median practitioner-to-population ratio or CSR across countries (country median). All three are normally reported for comparison. Risk ratios were calculated to test for a significant difference in proportions between 2011 and 2020 regional ratios.

Given that CSRs are affected by both surgeon-to-population ratios as well as by surgical efficiency ratios, 2020 CSRs were additionally predicted under two ideal scenarios (target ratio of surgeons achieved with current surgical efficiency maintained, and target surgical efficiency reached with current growth of surgeons maintained) to identify where investment might best be focused to achieve the 2,000 cataract surgeries per million population indicator in each country.

To explore associations between target ratios and eye health system structure, two national cataract surgeon variables were constructed: a categorical variable classifying countries with cataract surgeons (>1 practitioner) and ‘without’ (≤1 practitioners; in countries with only one cataract surgeon, a human resource strategy to train and employ this cadre was assumed not to be in place), compared using Wilcoxon rank sum tests; and a continuous variable estimating the proportion of all surgeons that cataract surgeons represent in the country, tested using Spearman rank correlations. Correlations between CSR and HReH ratios were also plotted and explored this way. Non-parametric tests were employed in the analysis because of the limited number of observations and because the data did not fit normal distributions.

### Ethics statement

This study was approved by the London School of Hygiene & Tropical Medicine’s Ethics Review Committee.

## Results

### Surgeons

**Figure 1:**
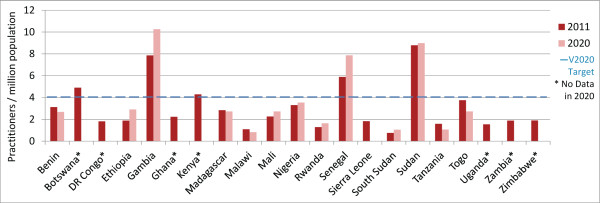
**Surgeons per million population in 2011 and 2020 (projected).** Complete national workforce entry and exit data needed to make year 2020 projections were limited and differed by cadre. Whereas 2011 ratios could be calculated for surgeons and ophthalmic clinical officers (OCOs)/nurses in 21 countries, and in 18 countries for refractionists, year 2020 ratios could only be calculated in 14 countries for surgeons, 15 for OCOs/nurses, and 7 for refractionists (16 countries in total contributed data for analysis of at least one cadre). Year 2020 cataract surgery rate projections were limited to the countries for which surgeon projections could be made. Data for 2011 are only presented here for countries in which projections could be made.

**Figure 2:**
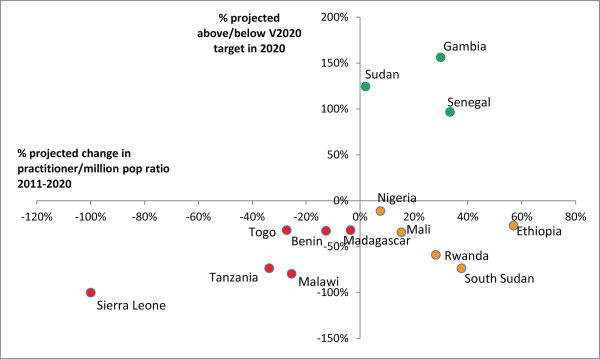
**VISION 2020 projected performance: surgeons ratio.** Countries in green are expected to be above target in 2020, countries in yellow are below target but their practitioner-to-population ratios are expected to increase, countries in red are below target and their ratios are expected to decrease. Insufficient information was available to identify whether ratios were increasing or decreasing in two countries currently above target (Botswana and Kenya) and five countries currently below (Democratic Republic of Congo, Ghana, Uganda, Zambia and Zimbabwe).

In 2011, 5/21 countries (Botswana, Gambia, Kenya, Senegal and Sudan) met or exceeded the VISION 2020 target ratio for surgeons (ophthalmologists and cataract surgeons combined) (Figure 1). Considering projection data available from 14 countries, practitioner-to-population ratios in this sample are expected to increase in more countries than they are expected to decrease (8 vs 6) (Figures 1 and 2, and Additional file 1 for results by country), with the 14-country ‘regional’ surgeon-to-population ratio predicted to increase slightly, from 3.1 in 2011 to 3.4 in 2020 (Table 2, difference in 2011 and 2020 regional proportions *P*-value 0.71, data not shown).

**Table 2:** Practitioners and surgeries per million population in 2011 and 2020 (projected), by cadre

**VISION 2020 indicator**		**2011**				**2020 (projected)**			
	**Number of countries included**	**Sub-Saharan Africa regional ratio**	**Country mean**	**Country median**	**(Min-max)**	**Sub-Saharan Africa regional ratio**	**Country mean**	**Country median**	**(Min-max)**
Ophthalmologists	14	**2.6**	2.2	1.2	(0.5–8.8)	**2.7**	2	1.5	(0–9.0)
Cataract surgeons	14	**0.4**	1.1	0.5	(0–7.3)	**0.7**	1.5	0.3	(0–9.8)
**All surgeons**	**14**	**3.1**	**3.3**	**2.5**	**(0.8–8.8)**	**3.4**	**3.5**	**2.7**	**(0–10.3)**
Ophthalmic clinical officers (OCOs)	15	**0.8**	1.1	0	(0–6.2)	**1.0**	1.6	0	(0–10.2)
Ophthalmic nurses	15	**5.0**	5.7	6.2	(0–22)	**5.8**	6.3	4.2	(0–37.5)
**All OCOs/nurses**	**15**	**5.8**	**6.8**	**6.2**	(0–22)	**6.8**	**7.9**	**6.4**	(0–37.5)
Optometrists	7	**2.9**	1.4	0.1	(0–8.8)	**2.4**	1.2	0.1	(0–7.1)
Mid-level refractionists	7	**0.7**	0.5	0.3	(0–1.5)	**1.1**	1.0	0.7	(0–3.3)
**All refractionists**	**7**	**3.6**	**1.9**	**0.4**	**(0.3–9.9)**	**3.6**	**2.3**	**1.1**	**(0.4–7.8)**
Cataract surgeries	14	**578**	731	521	(309–2,210)	**657**	824	476	(0–2,570)
Cataract surgeries per surgeon	14	**188**	255	254	(85–483)	**192**	246	251	(0–528)

Figures in bold indicate ratios for combined categories of practitioners (rows) or for the sub-Saharan Africa region (columns). All indicators are ratios of practitioners or surgeries per million population, except where noted. For further details of proportional population group in the region and by country, see Additional file 1.

These changes in practitioner-to-population ratios reflect relative changes in underlying practitioner and general population growth. Considering pooled data, between 2011 and 2020 the regional surgeon population is expected to increase by 41% (country range -100% to +129%) which will be greater than the predicted 27% general population growth during this period (country range +19% to +65%; 29% growth predicted in the population over 50 years of age); however, wide variation in projections between countries should be noted.

**Figure 3:**
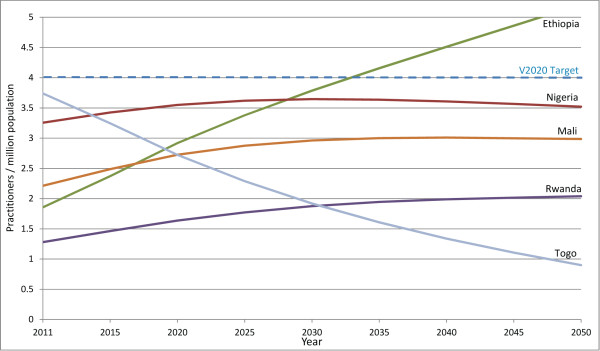
**Projected surgeon-to-population ratios 2011–2050 in five selected countries.** Projected surgeon-to-population ratios are presented for four countries that are currently below the VISION 2020 target but are projected to have increasing ratios between 2011 and 2020, and for one country (Togo) where the ratio is expected to decrease, even though it was close to meeting the VISION 2020 target in 2011. In three countries with increasing ratios, proportional practitioner population increases can eventually be expected to be outpaced by proportional general population increases and the VISION 2020 target will not be achieved without further intervention. Practitioner population growth is only expected to be rapid enough to replace the VISION 2020 practitioner shortage without intervention in Ethiopia by 2034.

Six countries already below target can expect a further decrease in their surgeon-to-population ratios either because more practitioners exit the workforce than enter (Sierra Leone, Tanzania and Togo), or because, although the practitioner population is increasing, it is increasing too slowly to account for growth in the general population (Benin, Madagascar and Malawi). Sierra Leone is expected to have no surgeons at all by 2020, assuming current (2008 to 2010) practitioner entry and exit rates, unless new initiatives are introduced to train or recruit more professionals.While five countries currently below target can expect to experience an increase in their surgeon-to-population ratios between 2011 and 2020 (Ethiopia, Mali, Nigeria, Rwanda and South Sudan), none are expected to reach the HReH target by the year 2020. In these countries, the number of surgeons is increasing and proportional practitioner growth is projected to be greater than growth in the general population between 2011 and 2020. If current practitioner population trends continue, in Ethiopia practitioner growth is expected to continue to rapidly outpace population growth after 2020, and the VISION 2020 target ratio will eventually be achieved by 2034, without further intervention (Figure 3). In all other countries surveyed, however, proportional practitioner population increases can eventually be expected to be outpaced by proportional general population increases before the target is reached. Therefore, the VISION 2020 target for surgeons will never be achieved in these countries without additional intervention, even in Nigeria which is expected to be close to the target in 2020 (Figure 3).

### Ophthalmic clinical officers/nurses

**Figure 4:**
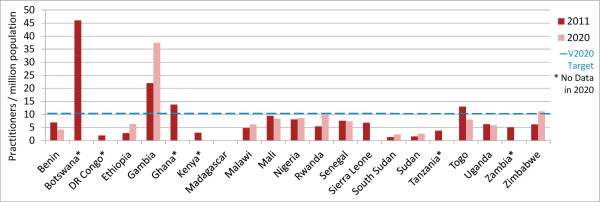
Ophthalmic clinical officers/nurses per million population in 2011 and 2020 (projected).

**Figure 5:**
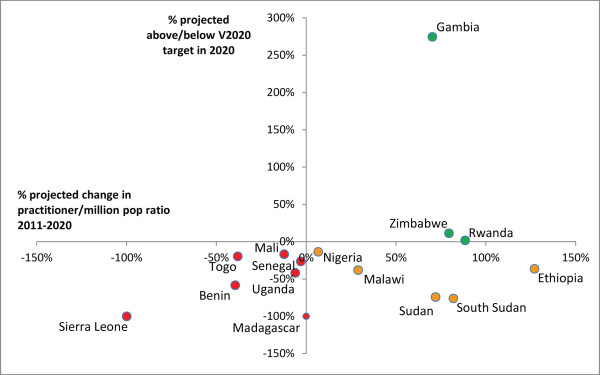
**VISION 2020 projected performance: Ophthalmic clinical officers/nurses ratio.** Countries in green are expected to be above target in 2020, countries in yellow are below target but their practitioner-to-population ratios are expected to increase, and countries in red are below target and their ratios are expected to decrease. Insufficient information was available to identify whether ratios were increasing or decreasing in two countries currently above target (Botswana and Ghana) and four countries currently below (Democratic Republic of Congo, Kenya, Tanzania and Zambia).

In 2011, 4/21 countries (Botswana, Gambia, Ghana and Togo) met or exceeded the VISION 2020 target ratio for OCOs/nurses. Practitioner-to-population ratios are expected to increase in more countries than they are expected to decrease (8 versus 7; country n = 15) (Figures 4 and 5), with the regional OCO/nurse-to-population ratio predicted to increase from 5.8 in 2011 to 6.8 in 2020 (Table 2, difference in 2011 and 2020 regional proportions *P*-value 0.37, data not shown).

**Figure 6:**
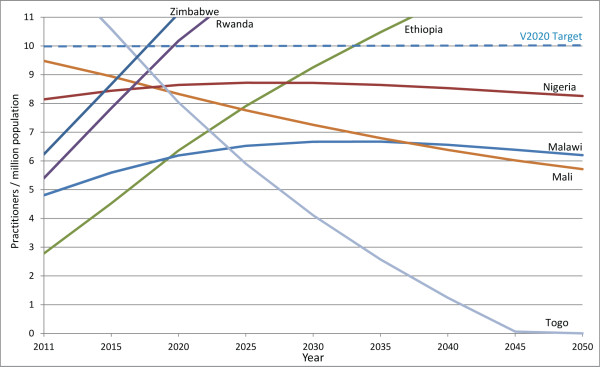
**Projected ophthalmic clinical officers/nurses-to-population ratios 2011–2050 in seven selected countries.** Projected ophthalmic clinical officers/nurse-to-population ratios are presented for five countries that are currently below the VISION 2020 target but are projected to have increasing ratios between 2011 and 2020, and for two countries (Mali and Togo) where the ratio is expected to decrease, even though it was above (Togo) or close to (Mali) meeting the VISION 2020 target in 2011. In two of the countries (Malawi and Nigeria) with increasing ratios, proportional practitioner population increases can eventually be expected to be outpaced by proportional general population increases and the VISION 2020 target will not be achieved without further intervention. Practitioner population growth is expected to be rapid enough to replace the VISION 2020 practitioner shortage without intervention in Zimbabwe and Rwanda before 2020 and in Ethiopia by 2034.

Between 2011 and 2020, the regional OCO/nurses-to-population ratio is expected to increase by 49% (country range -100% to +200%) which will be greater than the predicted 27% general population growth (country range +9% to +65%). Five countries already below target can expect a further decrease in their OCO/nurse-to-population ratios either because more practitioners exit the workforce than enter (Benin, Sierra Leone), or because, although the practitioner population is increasing, it is increasing too slowly to account for growth in the general population (Mali, Senegal, Uganda). Similar to surgeons, Sierra Leone is expected to have no OCOs or ophthalmic nurses at all by 2020, assuming current (2008 to 2010) practitioner entry and exit rates. Although Togo is currently above the VISION 2020 target for OCOs/nurses, current practitioner entry and exit trends predict that the country will actually fall below target by 2020.Seven countries below target in 2011 can expect to experience an increase in their OCO/nurse-to-population ratios between 2011 and 2020 (Ethiopia, Malawi, Nigeria, Rwanda, South Sudan, Sudan, Zimbabwe), with two (Rwanda and Zimbabwe) expected to reach the HReH target by the year 2020. In these countries, the number of OCOs/nurses is increasing and proportional practitioner growth is projected to be greater than growth in the general population between 2011 and 2020. As for surgeons, Ethiopia is also expected to achieve the VISION 2020 target ratio without further intervention by 2034 (Figure 6). In all other countries, however, proportional practitioner population increases can eventually be expected to be outpaced by proportional general population increases before the target is reached, without additional intervention.

### Refractionists

**Figure 7:**
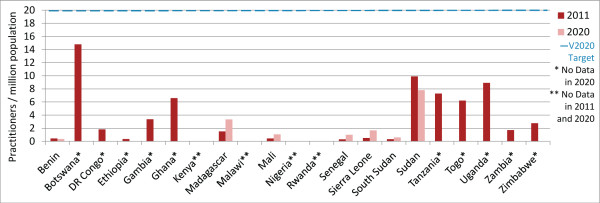
Refractionists per million population in 2011 and 2020 (projected).

**Figure 8:**
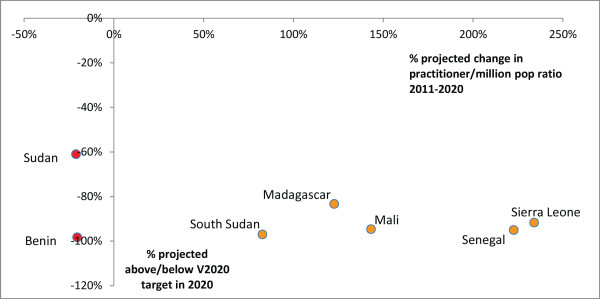
**VISION 2020 projected performance: refractionists ratio.** Countries in yellow are expected to be below target in 2020 but their practitioner-to-population ratios are expected to increase, and countries in red are below target and their ratios are expected to decrease. Insufficient information was available to identify whether ratios were increasing or decreasing in the remaining 14 countries currently below target.

In 2011, no countries (0/17) met the VISION 2020 target ratio for refractionists (optometrists and mid-level refractionists combined). Considering projection data from only seven countries, practitioner-to-population ratios are expected to increase in more countries than they are expected to decrease (5 versus 2) (Figures 7 and 8), but no change in the regional refractionist-to-population ratio is predicted between 2011 and 2020 (2011 and 2020 projected regional ratios both 3.6, Table 2).

Between 2011 and 2020, the regional refractionist population is expected to increase by 31% (country range 0% to +300%) which will be similar to the predicted 32% general population growth during this period (country range +20% to +65%). Two countries (Benin and Sudan) already below target can expect a further decrease in their refractionist-to-population ratios, in Benin because no practitioners are entering the workforce, and in Sudan because, although the practitioner population is increasing, it is increasing too slowly to account for growth in the general population. Five countries below target in 2011 can expect an increase in their refractionist-to-population ratio between 2011 and 2020 (Madagascar, Mali, Senegal, Sierra Leone and South Sudan), but none are expected to reach even a quarter of the HReH target by the year 2020 and none are expected to reach it after 2020 without further intervention.

### Cataract surgical rates

**Figure 9:**
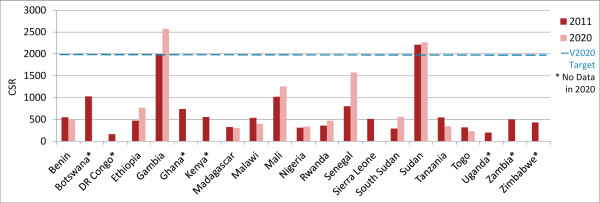
**Cataract surgeries per million population (CSR) in 2011 and 2020 (projected).** Estimates of surgeries for "2011" come from 2008 for Malawi, from 2010 for Benin, Botswana, Democratic Republic of Congo, Ethiopia, Gambia, Ghana, Kenya, Madagascar, Rwanda, Senegal, Sierra Leone, Sudan, Uganda and Zimbabwe, and from 2012 for South Sudan. The national estimate of 2011 cataract surgeries performed in Nigeria was based on proportional data provided from 13/37 states. In Botswana, surgeries data came from government facilities only.

**Figure 10:**
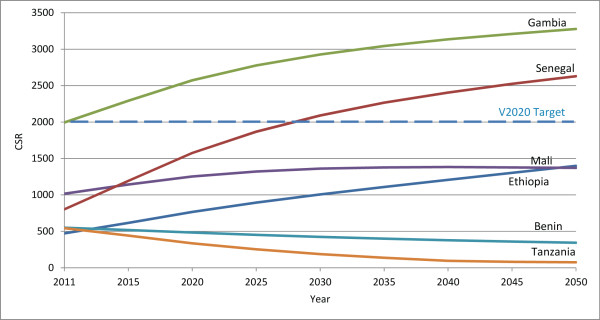
**Projected cataract surgery rates 2011–2050 in six selected countries.** Projected cataract surgery rates (CSRs) are presented for seven countries to illustrate different patterns of growth in the sample. Three countries that are currently below the VISION 2020 target are projected to have increasing ratios between 2011 and 2020. Senegal will eventually reach the VISION 2020 target without intervention in 2028, Ethiopia may reach the target after 2050, but in Mali, proportional growth in CSR will eventually be outpaced by growth in the general population and the VISION 2020 target is never expected to be achieved without intervention. Currently at the VISION 2020 target, Gambia’s CSR is expected to increase further, while the CSR in Benin and Tanzania, currently below target, is expected to decrease further, beyond 2020.

**Figure 11:**
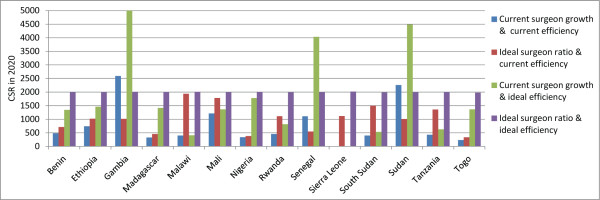
Projected cataract surgery rates (CSRs) in year 2020 by country, under different intervention scenarios.

In 2011, 2/21 countries (Gambia and Sudan) had met (or nearly met) the VISION 2020 CSR target of 2,000 surgeries per million population. CSRs are expected to increase in more countries than they are expected to decrease (8 versus 6, country n = 14) (Figure 9, Additional file 1), and the regional CSR is predicted to increase slightly, from 578 in 2011 to 657 in 2020 (Table 2, difference in 2011 and 2020 regional proportions *P*-value 0.03, data not shown). If only changes in human resource levels are considered, the regional ratio of surgeries performed per surgeon (surgical efficiency) would not be expected to appreciably increase in 2020 (14-country regional ratio 188 in 2011 versus 192 in 2020).Considering current growth trends for each surgical cadre, each cadre’s current surgical efficiency, and projected general population growth, between 2011 and 2020 the number of surgeries performed in the 14-country sample is expected to increase by 44% (country range -100% to +220%) which will be greater than the predicted 27% general population growth during this period. Six countries already below target can expect a further decrease in their CSR (Benin, Madagascar, Malawi, Sierra Leone, Tanzania and Togo). Because no surgeons are projected to be working in the year 2020 in Sierra Leone, the number of projected cataract surgeries is also projected to be zero. While six countries currently below target can expect to experience an increase in CSR between 2011 and 2020 (Ethiopia, Mali, Nigeria, Rwanda, Senegal and South Sudan), none are expected to reach the VISION 2020 target by the year 2020. Senegal is expected to reach this target by 2028, but no other countries will after 2020 without further intervention (Figure 10).In analyses of the year 2020 CSRs under two ideal scenarios (Figure 11), one country (Malawi) would nearly reach the CSR target if the target ratio of practitioners per population was achieved alone; another country (Senegal) would reach the target if only the surgical efficiency was improved. All other countries would require a combination of interventions, with an equal number benefiting more from a greater emphasis on increasing surgeon numbers (six countries: Malawi, Mali, Rwanda, Sierra Leone, South Sudan and Tanzania) or surgical efficiency (six countries: Benin, Ethiopia, Madagascar, Nigeria, Senegal and Togo).

### VISION 2020 target performance associated with cataract surgeon cadre

#### *Year 2011 performance*

**Figure 12:**
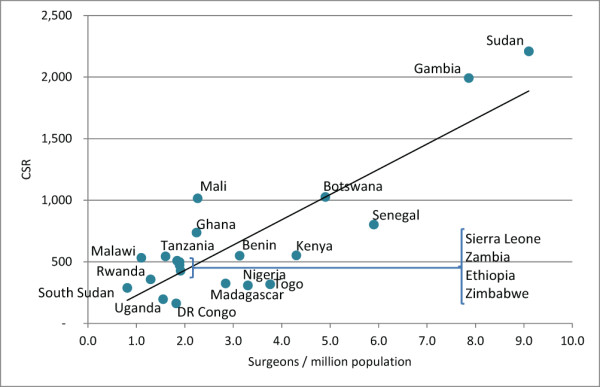
Relationship between surgeons-to-population ratio and cataract surgery rate (CSR) in 2011.

A positive association was found between 2011 combined surgeon practitioner ratios and CSRs (R = 0.6103, *P*-value 0.0031, Figure 12), meaning that countries with more surgeons per population (of any type) performed correspondingly more surgeries per population.

**Figure 13:**
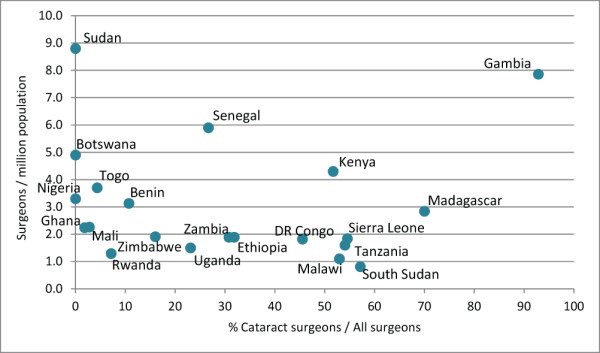
Relationship between surgeon-to-population ratio and proportional presence of cataract surgeons in workforce in 2011.

**Figure 14:**
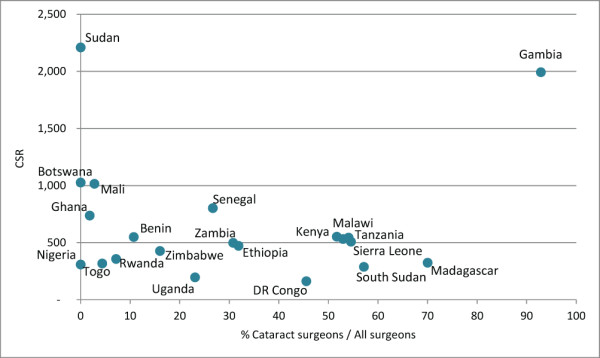
Relationship between cataract surgery rate (CSR) and proportional presence of cataract surgeons in workforce in 2011.

Seven out of 21 countries in the sample did not have cataract surgeons in their active workforce (≤1 practitioners). When compared to the rest of the sample, countries ‘without’ the cataract surgeon cadre appeared to have significantly higher surgeon-to-population ratios (*P* = 0.0014) and CSRs (*P* < 0.0001, Table 3). Dose–response relationships with the proportional representation of cataract surgeons in the surgeon workforce were not, however, observed (R = -0.3485, *P* = 0.12 and R = -0.188, *P* = 0.41 for the associations between the percentage of cataract surgeons out of all surgeons with surgeons per million population and CSR, respectively; Figures 13 and 14). Additionally, whether or not countries had cataract surgeons was not associated with the overall number of cataract surgeries performed per surgeon (surgical efficiency, *P* = 0.4594, Table 3).

**Table 3:** VISION 2020 target performance according to presence of cataract surgeon cadre in 2011

	**Countries with ≤1 CS (n = 7)**	**Countries with >1 CS (n = 14)**	***P*-value**
**Surgeons per million population**			
Regional ratio	3.2	1.1	**0.0014**
Country mean	3.8	2.7	
Country median	3.3	1.9	
**Cataract surgery rate**			
Regional ratio	654	419	**<0.0001**
Country mean	854	561	
Country median	738	504	
**Surgeries per surgeon**			
Regional ratio	174	186	0.4594
Country mean	243	230	
Country median	252	244	

CS, cataract surgeon. *P*-values in bold indicate a difference in proportions (risk ratios) significant at the 5% level.

#### *Projected practitioner growth 2011 to 2020*

Of five countries that met or exceeded the VISION 2020 surgeon-to-population ratio in 2011, two (Kenya and Gambia) would not have met this target if only ophthalmologists were considered. Although the surgeon-to-population target was met by ophthalmologists alone in Senegal in 2011, because of slow growth projected in this practitioner population compared to the general population ophthalmologists alone would not be expected to meet the target in 2020 (see Additional file 1).

When projected proportional practitioner growth in 14 countries is compared between the two cadres, higher practitioner population increases are expected for cataract surgeons (regional projected proportional growth +96%) than ophthalmologists (+30%), with notably fast growth in the cataract surgeon workforce in Senegal and Ethiopia. The addition of cataract surgeons in considerations of combined surgeon HReH target performance increases proportional practitioner population growth in 7/9 countries with this cadre (Additional file 1) and, importantly, brings practitioner growth higher than general population growth (necessary to observe an increase in practitioner-to-population ratio between 2011 and 2020) in three countries (South Sudan, Senegal and Gambia).

## Discussion

This study predicted the performance of eye health workforce development in 16 countries of sub-Saharan Africa by the year 2020, according to key HReH and service delivery indicators suggested by the global VISION 2020 programme. Using information on current workforce entry and exit dynamics and HReH structure, we built on our previous analysis of current national and regional performance which suggested that a minority of countries surveyed had achieved these VISION 2020 targets in 2011 (Palmer and colleagues, manuscript submitted). Although, across cadres, the eye health practitioner workforce appears to be growing in more countries than it is shrinking, based on current trends very few additional countries in our sample are expected to achieve VISION 2020 targets by year 2020 or after, without further intervention.

Across our analyses, in many ways, cataract surgeons seemed to perform as effectively as ophthalmologists in relation to achievement of VISION 2020 cataract surgery targets. Surgical rates per surgeon (considering both types) were similar among countries which did and did not employ this cadre, and countries with a higher proportion of cataract surgeons in their surgeon workforce did not have proportionately higher CSRs. Indeed, there seemed to be a very simple relationship between surgeons and CSR: the more surgeons a country had, the more surgeries were performed, regardless of the type of surgeon employed.

That countries with cataract surgeons, who are trained to specialise in this type of surgery, do not display higher CSRs is counter-intuitive and deserves further study to investigate the complex factors which underlie this finding. We could not, for example, verify that all surgeons (either cataract surgeons or ophthalmologists) reported in the 2011 active workforce were actually performing surgeries to assess efficiency of the surgically active workforce for either cadre, specifically. Cataract surgical performance in Africa varies widely between individual surgeons and has been associated with time since graduation, access to surgical equipment and working in the more highly resourced non-governmental or private sectors [6,7,12]. Additionally, contrary to our initial assumption, not all ophthalmologists in our sample can necessarily be assumed to have been trained in cataract surgery (Serge Resnikoff, personal communication). Our predictions of future CSR considered only future changes in workforce growth (based on current trends), but explored how CSRs could be improved under additional intervention scenarios. For most countries, a combination of interventions to improve both surgeon numbers and surgical efficiency of the workforce would be needed to achieve the VISION 2020 target. Naturally, neither statistic reflects an assessment of surgical quality of care, another essential component of any programme designed to treat or prevent visual impairment due to cataracts [2].

Equally counter-intuitive was our finding that countries without the cataract surgeon cadre (≤1 practitioner in the country) as a group had significantly higher ‘surgeon’ practitioner-to-population ratios (and therefore CSRs) than countries which have adopted this cadre. In fact, this might be expected if the introduction of this cadre has historically been viewed as an appropriate human resource strategy to adopt only in poorly performing countries (that is, cataract surgeons are ‘not needed’ in countries which already have sufficient ophthalmologists) or in poor countries (since human resource levels are commonly predicted by gross domestic product or national income [13] and cataract surgeons are cheaper to train and retain). We found that, in sub-Saharan Africa, wealthier countries tended to have higher HReH practitioner ratios, particularly for ophthalmologists (Palmer and colleagues, manuscript submitted). Comparable findings were identified in Latin America, where CSR has been positively correlated with national income [14].

It is possible that insufficient time has elapsed for the impact of such a task-shifting strategy to affect practitioner-to-population ratios in our sample. The contribution of the cataract surgeon cadre to achievement of the surgeon ratio in Gambia, the earliest adopter of this cadre in Africa, has already been noted. Regionally, the cataract surgeon workforce appears to be growing at a much faster rate than ophthalmologists (+96% versus +30% between 2011 and 2020, with the general population over 50 years of age growing at +29%) probably because they require less time and resources to train than ophthalmologists and are also probably easier to retain. Combined with the fact that cataract surgeons are much more likely to be employed in the public sector and in rural areas where practitioner ratios are lowest (Palmer and colleagues, manuscript submitted) and where elderly people are furthermore likely to live, this is a major advantage for countries wishing to improve their eye health system performance and equity. Apart from Ethiopia, which displayed a very high projected proportional increase from a moderately high base because of ever-increasing need for services in the growing general population, all other countries in our sample currently below target will need to substantially increase investment in their surgical workforce if they are ever to achieve the minimum HReH targets set by VISION 2020. Growth in global populations is mainly to blame for the relatively little change in global burden of eye disease between 1990 and 2010, despite two decades of eye health intervention [15]. Additionally, ‘minimum’ HReH targets for Africa may evolve as visual impairment due to the most easily preventable conditions such as cataract decreases across Africa; more ophthalmologists, in particular, will need to be trained in the medium-to-long term to manage the more complex interventions associated with glaucoma and diabetic retinopathy. Similar investigations into the role of mid-level refractionists in the optometry workforce and refractive service output (for example, using the indicator spectacles distributed or sold) may be warranted, but more robust data are also needed. Like others before us [16], we found these cadres to be the most difficult to collect data on, potentially because most refractionists work in the private sector which governments have less incentive to monitor (for further discussion, see Palmer and colleagues, manuscript submitted). Practitioner exit data, necessary to make reliable projections about practitioner population growth, were particularly difficult to locate and meant that we could only draw limited conclusions about ‘regional’ refractionist workforce development in sub-Sahara using data from seven countries. The contribution of ophthalmic nurses and TSOs to provision of refraction services also could not be systematically explored.

Finally, although data collection from the most populous countries in sub-Saharan Africa was prioritised, a paucity of easily available, routinely collected and reliable HReH and cataract data from the region means that our analyses are only truly representative of around a third (16/48) of countries in sub-Sahara; it remains to be seen how representative our findings are of trends in the wider region.

## Conclusions

The VISION 2020 campaign emphasises the key role that eye care human resources development plays in reducing vision loss globally and suggests key HReH indicators. To predict future VISION 2020 indicator achievement, national programmes need to monitor not only training programme graduate rates, but also rates of national workforce exit and how, when combined, these rates compare to growth in the general population in need of ophthalmic services. In our sample of a third of countries in sub-Saharan Africa, across cadres, the eye health practitioner workforce appears to be growing in more countries than it is shrinking. Apart from countries which have already achieved VISION 2020 indicator targets, however, very few additional countries are expected to achieve these by year 2020 or after without further intervention. Countries seeking to make rapid progress to improve CSR, in particular, could prioritise investment in new cataract surgeon over ophthalmologist training, since the cataract surgeon workforce can be expanded more quickly. Synergistic investment in understanding and addressing reasons why cataract surgeons appear not to be more productive than ophthalmologists in cataract surgical terms is also recommended.

## Abbreviations

CSR: cataract surgery rate; HReH: human resources for eye health; OCO: ophthalmic clinical officer; TSO: techniciens supérieurs en ophtalmologie; WHO: World Health Organization.

## Competing interests

The authors declare that they have no competing interests.

## Authors’ contributions

JP, SF, JJP, DPatel and KB designed the methodology and the data collection tools. JP, SF, DPillay and FC collected data. JP, FC and AG analysed data. JP drafted the article. JJP, KN RG, DPatel and KB reviewed the draft version. All authors read and approved the final manuscript.

## Authors’ information

JJP, MSc, PhD, is a Research Fellow at the International Centre for eye Health in the Clinical Research Department at the London School of Hygiene & Tropical Medicine.

FC, BSc, MPhil, is the Statistician for the African Vision Research Institute and the Brien Holden Vision Institute.

AG, BSc, MSc, was a Research Assistant at the International Centre for eye Health in the Clinical Research Department at the London School of Hygiene & Tropical Medicine.

DPillay, IMM Dip (Marketing Management), was the African Vision Research Institute Research Co-ordinator on the study.

SF, BSc, MSc, is a Public Health Registrar and was a Research Assistant at the International Centre for eye Health in the Clinical Research Department at the London School of Hygiene & Tropical Medicine.

JJP, MA, PhD, is the Research Manager of the African Vision Research Institute.

KN, OD, MPh, PhD, is the CEO of the African Vision Research Institute, the Global Programmes Director of the Brien Holden Vision Institute and the Africa Chair of the International Agency for the Prevention of Blindness.

RG, MA, Dip. Ed., MA, is the Director of HRH Programmes, IAPB Africa.

DPatel, MD, MMed (Ophth), MSc (CEH), is a Lecturer in Public Health Ophthalmology at the International Centre for Eye Health in the Clinical Research Department at the London School of Hygiene & Tropical Medicine.

KB, MMgt, MScPH, PhD, is a Lecturer in health systems research at the International Centre for Eye Health in the Clinical Research Department at the London School of Hygiene & Tropical Medicine.

## Supplementary Material

Additional file 1 **Practitioners and surgeries per million population in 2011 and 2020 (projected), by cadre and by country.** Figures in bold indicate ratios for combined categories of practitioners (columns) or for the sub-Saharan Africa region (rows). When the projected proportional change in a practitioner population (or number of surgeries performed) is greater than the proportional change projected for the general population, an increase in the practitioner-to-population ratio (or cataract surgery rate) by 2020 can be expected. Click here for file

## References

[B1] V2020 Global Initiative for the Elimination of Avoidable Blindness: Action Plan 2006–2011 2007 Geneva: World Health Organization [http://www.who.int/blindness/Vision2020_report.pdf]

[B2] WHO Action Plan for the Prevention of Avoidable Blindness and Visual Impairment 2014–2019 2013 Geneva: World Health Organization [http://www.who.int/blindness/actionplan/en/]

[B3] Raman U Human resources for eye care: changing the way we think Community Eye Health J 2009 22 12 PMC2683557 19506715

[B4] IAPB Joint Report of the HReH Task Team & of the Africa Wide Workshop on HReH: Past, Present and Future 2013 London: International Agency for the Prevention of Blindness

[B5] Lewallen S Etya'ale D Kello AB Courtright P Non-Physician Cataract Surgeons in Sub-Saharan Africa: Situation Analysis 2012 London: TM & IH: Tropical medicine & international health 10.1111/j.1365-3156.2012.03084.x 22973827

[B6] Hale I Lewallen S Courtright P Task-Shifting: Ophthalmologist to Non-Physician Cataract Surgeon: a Review of the Evidence 2013 Moshi, Tanzania: Kilimanjaro Centre for Community Ophthalmology (KCCO) [http://www.iapbafrica.co.za/resource/resourceitem/206/1]

[B7] Courtright P Ndegwa L Msosa J Banzi J Use of our existing eye care human resources: assessment of the productivity of cataract surgeons trained in eastern Africa Arch Ophthalmol 2007 125 684 687 17502509 10.1001/archopht.125.5.684

[B8] Resnikoff S Felch W Gauthier TM Spivey B The number of ophthalmologists in practice and training worldwide: a growing gap despite more than 200,000 practitioners Br J Ophthalmol 2012 96 783 787 10.1136/bjophthalmol-2011-301378 22452836

[B9] Fapohunda B Fronczak N Minichiello SN Buckner B Schenck-Yglesias C Bates C Patil P Dal Poz M, Gupta N, Quain E, Soucat ALB Use of facility-based assessments in health workforce analysis Handbook on Monitoring and Evaluation of Human Resources for Health with special applications for low- and middle-income countries 2009 Geneva: World Health Organisation

[B10] WHO Mental Health Systems in Selected Low- and Middle-Income Countries: a WHO-AIMS Cross-National Analysis 2009 Geneva: World Health Organisation [http://www.who.int/mental_health/evidence/who_aims_report_final.pdf]

[B11] Probabilistic Population Projections based on the 2010 Revision of the World Population Prospects [http://esa.un.org/unpd/ppp/Data-Output/UN_PPP2010_output-data.htm]

[B12] Habtamu E Eshete Z Burton MJ Cataract surgery in Southern Ethiopia: distribution, rates and determinants of service provision BMC Health Serv Res 2013 13 480 10.1186/1472-6963-13-480 24245754 PMC3842739

[B13] Scheffler RM Liu JX Kinfu Y Dal Poz MR Forecasting the global shortage of physicians: an economic- and needs-based approach Bull World Health Organ 2008 86 516B 523B 10.2471/BLT.07.046474 18670663 PMC2647492

[B14] Lansingh VC Resnikoff S Tingley-Kelley K Nano ME Martens M Silva JC Duerksen R Carter MJ Cataract surgery rates in Latin America: a four-year longitudinal study of 19 countries Ophthalmic Epidemiol 2010 17 75 81 10.3109/09286581003624962 20302429

[B15] Stevens GA White RA Flaxman SR Price H Jonas JB Keeffe J Leasher J Naidoo K Pesudovs K Resnikoff S Taylor H Bourne RR Vision Loss Expert Group Global prevalence of vision impairment and blindness: magnitude and temporal trends, 1990–2010 Ophthalmology 2013 120 2377 2384 10.1016/j.ophtha.2013.05.025 23850093

[B16] V2020 Global Human Resource Development Assessment for Comprehensive Eye Care 2006 London: Vision2020 Human Resources Development Working Group, Pakistan Institute of Community Ophthalmology [http://www.iapb.org/sites/iapb.org/files/Global_HR_Development_Assessment.pdf]

